# Supplementary material for: The ribosome lowers the entropic penalty of protein folding
Source: Nature. 2024 Aug 7;633(8028):232–9. doi: 10.1038/s41586-024-07784-4 (PMC11374706; doi:10.1038/s41586-024-07784-4)
Supplement: Supplementary file 1 — Description of additional analyses as supplementary notes, including methodological background and quality control for PRE-NMR experiments, comparison of molecular dynamics force fields, validation of molecular dynamics simulations against experimental data, convergence of molecular dynamics simulations, and a description of additional energetic analyses. The Supplementary Information also contains supplementary tables and figures. [file 41586_2024_7784_MOESM1_ESM.pdf]

nature portfolio

https://doi.org/10.1038/s41586-024-07784-4

Supplementary information

The ribosome lowers the entropic penalty of protein folding

In the format provided by the authors and unedited

Supplementary information

Note 1: PRE-NMR experiments & MTSL labelling

We recorded paramagnetic relaxation enhancement (PRE) NMR experiments of FLN5 A_3A_3 on and off the ribosome (**Extended Data Fig. 1B-D**). PREs provide a sensitive measure of long-range distances (10-25Å) within disordered proteins [106,143-145]. PREs were measured using the ^{1}H,^{15}N-correlated NMR signal intensities of the paramagnetic state resonances relative to those recorded under diamagnetic (i.e., reducing) conditions and plotted along the protein sequence (**Extended Data Fig. 1C-D,** see **Supplementary Figure 1** for full spectra). Six labelling sites within the FLN5 sequence (without the native cysteine, C747V), were chosen for MTSL labelling, focusing on the C-terminal region (N730-K746) that interacts with the ribosome [61] (**Extended Data Fig. 1B**).

For the isolated protein, short-range effects (~15-30 residues from the labelling site) are observed, suggesting compaction (**Extended Data Fig. 1D).** Moreover, long-range effects (sequence-specific broadenings) are observed along the polypeptide chain in regions distal to the labelling sites (**Extended Data Fig. 1D**), including within N-terminal residues A665-K680 (in the C657 dataset), and C-terminal residues D720-I738 (C699 & C706 datasets). Additionally, broadening is observed consistently around G700 (C734, C740 and C744 datasets) (**Extended Data Fig. 1D**). The same six cysteine variants (and two additional sites, **Extended Data Fig. 1D-F**) were then spin-labelled as RNCs. Several sites located near the C-terminus act as probes to the ribosome-interacting segment (residues N730-K746) [7,11] of FLN5 (**Extended Data Fig. 1D**, shaded area) whose resonances are broadened beyond detection (even in the absence of the spin label) [7].

The PRE profiles for each of the spin-labelled RNCs generally show broadening of resonances in the vicinity of the labelling sites, with few indications of long-range effects (**Extended Data Fig. 1D, Supplementary Figure 1**). Interestingly, despite being tethered to the ribosome, which is expected to slow tumbling of the protein [25,146] and therefore increase the extent of broadening due to the PRE effect [147], the RNC shows reduced broadening relative to the isolated protein in all datasets (**Extended Data Fig. 1D**). See supplementary note 2 for further details. This observation is made for all labelling sites, with a consistent trend across the protein sequence. The differences between the isolated and RNC samples are particularly apparent for dataset C740 and C744.

Intermolecular PREs between the NC and ribosome surface were measured to obtain additional distance restraints informing on the vicinity of the NC to the ribosome surface. They were obtained via spin-labelled RNC variants in which single cysteines had been introduced onto the surface of the ribosome in either the solvent-exposed uL24 loop (N53C) or C-terminal tail of uL23 (G90C) near the exit tunnel (**Extended Data Fig. 1E**) using CRISPR-Cas9 [2]. A greater extent of broadening in the uL23 dataset shows the NC's N-terminal preference to interact with regions near uL23 upon emerging from the exit tunnel (**Extended Data Fig. 1F**). Collectively, the experimental PRE data show that the unfolded state exhibits major differences in the conformations on the ribosome, appears to be more expanded

with fewer intramolecular contacts, and exhibits orientational preferences upon emerging from the tunnel.

All samples for PRE experiments were produced with uniform ^{15}N isotopic labelling in *E.coli* [7,11,61], purified to homogeneity, and spin-labelled with MTSL; the latter and sample integrity were confirmed using polyacrylamide gel electrophoresis, mass spectrometry, and interleaved NMR translational diffusion measurements (see **Extended Data Fig. 2A-E,H**). Complete spin-labelling of the isolated protein was confirmed using mass spectrometry, showing >98% labelling for two tested labelling sites (**Extended Data Fig. 2A-B).** For the RNC, MTSL labelling of the NC was qualitatively confirmed using a fluorescent MTSL analogue and in-gel fluorescence (see Methods, **Extended Data Fig. 2C**). In the NMR spectra, labelling sites C657, C699, C706, and C720 exhibited complete local broadening around the labelling site, suggesting that labelling was essentially complete. We also verified, using a cysteine mass-tagging and western blot approach [7], that the proximity of NC cysteines to the ribosome surface does not impede its accessibility for MTSL labelling. Cysteines placed at C699 and C740 show indistinguishable reactivity to a maleimide tag (**Extended Data Fig. 2D**). Labelling of engineered cysteines on the ribosome surface was confirmed using fluorescein-maleimide and in-gel fluorescence (**Extended Data Fig. 2E**). As RNC labelling was accompanied by background labelling of several ribosomal proteins (**Extended Data Fig. 2C**), we measured PREs in a cysteine-free FLN5+31 A_3A_3 variant, C747V (ΔCys) and observed no PRE effects in the NC (**Extended Data Fig. 2F**). This result suggests that non-specific spin-labelled ribosomal proteins and intermolecular effects between RNC molecules at this concentration (~10μM) do not contribute any PRE effects and thus any PREs observed in the RNCs originate exclusively from the introduced cysteine.

Note 2: Impact of slower dynamics on the measured PRE-NMR intensity ratios

The PRE-NMR data of the isolated and RNC constructs indicate that the structural ensemble on the ribosome is more expanded structurally (i.e., has a larger average R_g). The comparison between the data of the protein on and off the ribosome is, however, complicated by the fact that PRE-NMR intensity ratios depend both on the distance distributions and dynamics within the protein. Due to being tethered to the large ribosomal particle and ribosome surface interactions, the NC experiences slower dynamics particularly towards the C-terminus (higher τ_C values, **Supplementary Tables 5-6**), as indicated by reduced NMR signal intensities [7,12]. We therefore explored the impact of these increased dynamics by performing additional experiments of isolated FLN5 A_3A_3 (labelled at position C740) in different concentrations of glycerol. We reasoned that this would globally (across the sequence) lead to slower dynamics without changing the ensemble properties and distance distributions significantly. Indeed, we measured the diffusion coefficients of the protein under all conditions (0%, 5% and 18% glycerol) and found that its diffusion and radius of hydration does not change significantly, accounting for viscosity changes by diffusion measurements of DSS (**Extended Data Fig. 2J**). The PRE-NMR intensity ratios, however, showed that with increasing glycerol concentrations the intensity ratios become lower across the entire sequence (**Extended Data Fig. 2K**). This is in line with the expected PRE-NMR profiles for the same ensemble with slower dynamics (a higher τ_C uniform value, **Extended Data Fig. 2L**). On the

ribosome, a similar effect would be expected although with greater magnitude towards the C-terminus where tethering will restrict dynamics more significantly (**Extended Data Fig. 2M).** These experiments and calculations show that if the ensemble on the ribosome had the same distance distributions (and R_g) the PRE-NMR intensity ratios would be expected to be lower than those of the isolated protein. However, we observe for all labelling sites that the intensity ratios are higher on the ribosome, and this increase cannot be explained by dynamics (as dynamics would have the opposite effect). The higher PRE-NMR intensity ratios on the ribosome compared to in isolation thus support the notion that the chain is structurally expanded on the ribosome. Similarly, the higher PRE-NMR intensity ratios at short RNC length (FLN5+31) compared to longer lengths (FLN5+47 and FLN5+67) therefore supports the notion that the NC becomes less expanded as the ribosome elongates the polypeptide.

Note 3: Calculation of PRE rates for Bayesian reweighting

To quantify the transverse PRE relaxation rates (Γ_2) from intensity ratios, the approach outlined for ^{1}H-^{15}N HSQC [104,148] experiments was adjusted to account for the effect of ^{1}H–^{1}H couplings active during the multiple-quantum evolution period (t_1) in the ^{1}H-^{15}N SOFAST-HMQC experiment as following:

$$I_{dia} \propto \frac{1}{R_{2H}^{dia}\ x\ R_{2MQ}^{dia}}\, e^{-R_{2H}^{dia}\Delta} \qquad I_{para} \propto \frac{1}{R_{2H}^{para}\ x\ R_{2MQ}^{para}}\, e^{-R_{2H}^{para}\Delta} \qquad \text{Eq. S31}$$

where I_{dia} and I_{para} are the peak intensities (heights) of paramagnetic and diamagnetic respectively, Δ is the total time during which ^{1}H relaxation occurs (10.9 ms), and R_2^{dia} and R_2^{para} are the transverse relaxation rates for paramagnetic and diamagnetic amide protons, respectively. The $R_{2,H}$ and $R_{2,MQ}$ components of R_2^{dia} and R_2^{para} were calculated by multiplying the linewidths (LW) from the first and second dimension of the ^{1}H-^{15}N SOFAST HMQC spectra by π:

$$R_{2,H} = LW_{1_H}\ x\ \pi \qquad R_{2,MQ} = LW_{15_N}\ x\ \pi \qquad \text{Eq. S32}$$

The total electron spin-enhanced relaxation rate R_2^{para} is the sum of the intrinsic R_2^{dia} and spin contribution Γ_2 [149]:

$$R_2^{para} = R_2^{dia} + \Gamma_2 \qquad \text{Eq. S33}$$

After substituting equation S33 into equation S31, the intensity ratio for a particular amide proton is given by:

$$\frac{I_{para}}{I_{dia}} = \left(\frac{R_{2H}^{dia}}{R_{2H}^{dia}+\Gamma_2} \times \frac{R_{2MQ}^{dia}}{R_{2MQ}^{dia}+\Gamma_2}\right) e^{-\Gamma_2\Delta} \qquad \text{Eq. S34}$$

The transverse PRE relaxation rates (Γ_2) can be obtained by numerically solving equation S34 (performed in MATLAB).

Note 4: Classification of PRE restraints for Bayesian reweighting

Four classes of PRE transverse relaxation rates (Γ_2) were used for Bayesian reweighting. Peaks with intensity ratios between 0.2-0.8 were restrained as the calculated Γ_2 with propagated upper and lower bound relative errors derived as standard deviation of the spectral noise. Peaks with intensity ratios between 0.1-0.2 and 0.8-0.9 were restrained in a similar manner with an addition of 4% (isolated FLN5 A_3A_3) and 5% (FLN5 A_3A_3 RNC) propagated lower and upper bound errors. Peaks with an intensity ratio >0.9 were restrained only with an upper bound of 2.19 s^{-1} (isolated FLN5 A_3A_3) and 3.65 s^{-1} (FLN5 A_3A_3 RNC); peaks with intensity ratios <0.1 were restrained only with a lower bound of 64.48 s^{-1} (isolated FLN5 A_3A_3) and 96.02 s^{-1} (FLN5 A_3A_3 RNC).

Note 5: Comparison of force fields for isolated FLN5 A_3A_3

To obtain a high-resolution structural ensemble describing the unfolded FLN5 A_3A_3 variant, we obtained four different initial ensembles generated by different force fields. Three ensembles were generated by all-atom MD simulations in explicit water and one ensemble was generated using a coarse-grained model (referred to as “CA”, see methods). For all-atom simulations, two ensembles were simulated using the recently developed CHARMM36m (C36m) force field [86], which was optimised to yield better descriptions of both folded and unfolded proteins. As the authors pointed out in their paper, for some IDPs the C36m force field still yields ensembles that are more compact than expected from experiments [86], and it was proposed to use a modified water model where the water hydrogen Lennard-Jones well depth was increased, yielding more favourable protein-water dispersion interactions. Here, we test this possibility for our system by running simulations with the original CHARMM TIP3P water model (C36m) as well as this modified water model (referred to as C36m+W in this work). The third all-atom ensemble was obtained using the a99sb-disp force field, which was recently developed also to result in state-of-the-art descriptions of both folded and disordered proteins [87]. We ran a total of 10μs of unbiased MD simulations for all all-atom force field and water model combinations.

All ensembles, except the C36m ensemble, have similar distribution of radius of gyration (R_g) (**Extended Data Fig. 3A**). Consistent with previous reports [86,150], we observe a large increase in R_g for C36m+W compared to C36m. C36m generated the most compact ensemble (19.6 ± 0.4Å R_g) while C36m+W yielded the most expanded ensemble (30.3 ± 1.3Å R_g). The a99sb-disp and CA ensembles are slightly more compact compared to C36m+W with R_g values of 28.1 ± 0.9Å and 28.0 ± 0.1Å, respectively. We also analysed the fraction of native contacts relative to natively folded FLN5 (i.e., based on the crystal structure [94] PDB ID 1QFH). We find that all ensembles sample very few native contacts and have on average 2-4% of native contacts present (**Extended Data Fig. 3B-C**). The ensembles also differ substantially in the observed secondary structure propensities (**Extended Data Fig. 3C-D**). While the CA model predominantly samples coil structures as expected (99.1%), the all-atom MD ensembles have residual secondary structure to various degrees. The a99sb-disp ensembles samples the most helical structure (6.7 ± 0.7%), particularly at the N-terminus, in the D- and F-strand regions, as well as the E-F loop (Figure 1C-D). C36m on the other hand samples the most β-strand

structures (9.5 ± 1.3%), predominantly at the N-terminus, strands A', C, C', D, F, and G. These secondary structure preferences match the native secondary structure propensities surprisingly well. The C36m+W ensembles has preferences for β-strand structures in similar regions, but to a lesser extent overall (total 4.1 ± 0.5%), in agreement with its lower compactness compared to C36m. Finally, we analysed the inter-residue contact probabilities of the different ensembles. This analysis shows that C36m samples the most inter-residue contacts (**Extended Data Fig. 3E**), likely due to its higher compactness, whereas C36m+W and a99sb-disp have weaker contact preferences. Overall, the all-atom MD ensembles appear to substantially sample non-native contacts (i.e., outside the black contours which represent the native contact map). However, some native-like contacts are indeed also present. The C36m ensembles samples contacts between strands D&E, C&F and F&G, while the C36m+W ensembles contains contacts between strands B&E and C&F, but with weaker propensity. The a99sb-disp ensemble only samples native-like contacts between strands D&E and appears to be the most non-native. The CA ensembles, contrary to the others, samples barely any long-range contacts with most contacts near the diagonal, mainly at the C-terminus (**Extended Data Fig. 3E**).

We then proceeded to refine the isolated ensembles using a Bayesian inference (reweighting) approach[118]. As expected, the agreement with the experimental data increases dramatically in all cases (**Extended Data Fig. 3F-I**). However, the C36m ensemble shows significant local discrepancies, particularly near the labelling sites resulting in a lower fraction of frames contributing to the ensemble average after reweighting (N_{eff}, 0.11) and worse overall agreement with the PRE data (χ^2, 25.05) compared to the other ensembles (**Extended Data Fig. 3F-I**). This suggests that this prior ensemble contained few structural states that can explain the experimental data and significant modification to the prior is necessary. The local disagreement near the labelling sites is indicative of a rigidified backbone in these regions, potentially due to sampling overly compact structures. Indeed, the C36m ensemble is by far the most compact (average R_g of 19.6 ± 0.4 Å). The C36m+W and a99sb-disp are modified to a similar extent with a fraction of frames of 0.50 and 0.52, respectively. Their agreement with the PRE data is considerably better compared to the C36m ensemble, achieving values of χ^2 of 3.85 and 2.35 for C36m+W and a99sb-disp, respectively. This is broadly consistent with these ensembles sampling fewer compact states compared to C36m. Lastly, the CA ensembles achieved the highest N_{eff} and the lowest χ^2 value (0.83 and 1.61, respectively). Overall, C36m+W, a99sb-disp and the CA ensembles reach reasonable agreement with the PRE data, without excessive amounts of fitting, while the C36m ensemble appears to be a poor prior ensemble. In all cases, a balance between the force field and experimental is chosen using an L-curve analysis (**Extended Data Fig. 3I**).

Note 6: Comparison of isolated FLN5 A_3A_3 ensembles with validation data

We first assessed the agreement with the experimental radius of hydration (R_h) obtained from pulse-field gradient (PFG) NMR. This experiment provides a global measure of the compactness of the unfolded state. Compared to the experimental R_h, the C36m ensemble shows the largest discrepancy with an average R_h of 23.3 ± 1.2 Å and 25.2 ± 1.3 Å before and after reweighting, respectively

(**Extended Data Fig. 4A-B**). Thus, reweighting with the PRE data has led to an improvement in the agreement with R_h, but not sufficiently likely due to sampling predominantly compact states (**Extended Data Fig. 3A**). The C36m+W ensemble, on the other hand, has the best agreement with the experimental R_h before and after reweighting, with ensemble averages of 28.7 ± 1.4 Å and 30.6 ± 1.5 Å, respectively (**Extended Data Fig. 4A-B**). Therefore, reweighting with PRE data has also improved the agreement of this ensemble to almost within uncertainty (which comes predominantly from the forward model used to calculate R_h, see methods). For the a99sb-disp and CA ensembles the agreement also improves slightly after reweighting, however the change in R_h is smaller and yields ensembles averages less consistent than that of C36m+W with values of 28.7 ± 1.4 Å and 28.1 ± 1.4 Å, respectively. Overall, the posterior C36m+W ensemble therefore appears to best capture the average compactness (**Extended Data Fig. 4B**).

The same conclusion is reached when the ensembles are compared with small angle X-ray scattering (SAXS) data. The SAXS experimental data show that FLN5 A_3A_3 in isolation has an average R_g of 34.4 ± 0.6 Å by Guinier analysis (**Extended Data Fig. 4F-H**), and the R_g estimate is robust to the choice of method (we obtained 34.8 ± 0.1 Å by molecular form factor analysis[140], **Extended Data Fig. 4H**). The C36m ensemble also shows the largest discrepancy in this case, with an average R_g of 19.6 ± 0.4 and 23.1 ± 0.7 Å before and after reweighting, respectively. The a99sb-disp and CA ensembles agree better with the SAXS-derived R_g value prior to reweighting with average R_g values of 28.1 ± 0.9 Å and 28.0 ± 0.1 Å, respectively. Their agreement further improves slightly after reweighting with PRE-NMR data resulting in average R_g values of 29.5 ± 1.1 and 28.3 ± 0.1 Å, respectively. Consistent with the R_h analysis, however, the C36m+W ensemble agrees best with the SAXS-derived R_g value with an average of 30.3 ± 1.3 and 34.7 ± 1.1 Å before and after reweighting, respectively. We also compared the back-calculated SAXS profiles from the MD ensembles with the experimental profile (**Extended Data Fig. 4I**). The same trend is observed with this analysis, showing that the C36m+W ensemble has the lowest reduced χ^2 before and after reweighting. This analysis also validates that reweighting with the PRE-NMR restraints improves the quality of the ensembles, as for all force fields we observe improved agreement with the SAXS profile when reweighted (**Extended Data Fig. 4I**). In particular, the C36m+W ensemble exhibits a remarkable agreement with the experimental profile after reweighting (χ^2 = 0.7), supporting the accuracy of this force field when refined with PRE-NMR data.

When we compare the experimental chemical shifts with our MD ensembles, we find as in the case with R_h and SAXS, the C36m+W ensemble is in best agreement with all chemical shifts on average (**Extended Data Fig. 4D**). Indeed, the average RMSD for each nucleus is within the uncertainty of the forward model, suggesting that the ensemble captures the local and secondary structure preferences very well. This is both the case before and after reweighting, meaning reweighting does not substantially affect the ensemble-averaged chemical shifts. This is not surprising as chemical shifts report on very different phenomena than PRE experiments. However, in the case of the C36m ensembles, which did not agree well with PREs and R_h after reweighting, we find a modest but consistent worsening in the agreement with experimental shifts for all nuclei (**Extended Data Fig. 4D**), suggesting the comparison

with chemical shifts comparisons can in some cases still detect scenarios of poor prior ensembles and overfitting. The a99sb-disp ensemble is in worse agreement with the HN, N and Cα chemical shifts compared to the two C36m and C36m+W ensembles, but in better agreement with the C' chemical shifts (**Extended Data Fig. 4D**). Finally, the CA ensemble is in worst agreement with chemical shifts overall (**Extended Data Fig. 4D**). It is not unexpected that this ensemble does not agree very well overall with chemical shifts due to the nature of the simple force field and approximations made during all-atom back-mapping [96], but also shows that an ensemble without any secondary structure preferences agrees less well with chemical shifts. This ultimately supports that an ensemble with small populations of residual β-strand structure (as predicted by C36m+W) is the most consistent prediction with respect to the experimental NMR chemical shifts. We also independently verified that FLN5 A_3A_3 does not sample significant amounts of α-helix or β-sheet structure using circular dichroism (CD) spectroscopy. The CD spectrum exhibits a strong minimum at 198nm and low amplitude between 210-230nm, characteristic of a fully disordered protein (**Extended Data Fig. 4J**). Moreover, we estimated the secondary structure populations from the experimental NMR chemical shifts using δ2C [141]. These predictions showed that FLN5 A_3A_3 is predominantly coil and only samples very small amounts of residual α-helical or β-sheet structure, with a small preference for β-sheet structure, as observed with the C36m and C36m+W ensembles (**Extended Data Fig. 4K**).

Lastly, we compared our MD ensembles with the experimental backbone RDCs measured in PEG/octanol. Strikingly, we find that again the C36m+W ensemble is in better agreement than the other three ensembles (**Extended Data Fig. 4E**), consistent with the trend observed for the R_h, SAXS and chemical shift analysis. The C36m+W has a Q-factor 0.55 after reweighting with PREs, while the other ensembles Q-factors of 0.65-0.75. This level of agreement is in the range expected for disordered proteins [87]. Qualitatively, the C36m+W ensemble captures the trend of the experimental profiles well, particularly the $^1D_{NH}$ and $^1D_{C\alpha H\alpha}$ RDCs (**Extended Data Fig. 4E**). The effect of reweighting on the RDC profiles is minor.

In summary, comparison with our validation data has consistently revealed that the ensemble obtained with the C36m+W force field is the most consistent with other experimental data before and after reweighting with the PRE data. While a99sb-disp and CA also achieve good agreement with the PRE data before and after reweighting, their agreement with orthogonal data (particularly SAXS and RDCs) is worse than with C36m+W. This is, at least in part, due balancing force field and experimental data (**Extended Data Fig. 3I**) and we have also not explored using SAXS or RDCs as reweighting restraints. However, our analysis suggests that for the combination of C36m+W with PRE-NMR data as restraints results in the most accurate structural ensemble for this system.

Note 7: Convergence of isolated FLN5 A_3A_3 simulations using C36m+W

Given the C36m+W ensemble appeared to agree best with the experimental R_h, chemical shifts and RDCs, we appended the C36m+W simulation by running an additional 5x2μs from new starting

structures resulting in an ensemble worth 20μs of total sampling. It was then assessed whether the properties observed in the two sets of 10μs ensembles and the full 20μs ensemble showed consistent behaviour including agreement with experimental data. Convergence of MD simulations describing disordered proteins is highly challenging [151], but we are interested in whether the main conclusions and structural properties remain qualitatively consistent.

Prior to reweighting, all ensembles showed similar global structural properties including R_g, Q and secondary structure (**Supplementary Figure 5A-E**). We then reweighted both the first and second set of 10μs ensemble and the full 20μs ensemble using the PRE data (**Supplementary Figure 5J-L**). The PRE profiles were similar before reweighting and as expected even more after reweighting (**Supplementary Figure 5K-L**). The agreement with the validation data also remained highly similar (**Supplementary Figure 5M-O**), with all C36m+W ensembles being in better agreement with all the validation data compared to the other force fields. This also suggests that the structural properties captured by the validation data appear to be reasonably converged. Upon inspection of the ensemble structural properties, we find that after reweighting the ensembles from 10μs and 20μs of sampling are more similar than before reweighting with respect to global properties (R_g and Q), secondary structure and long-range contacts (**Supplementary Figure 5F-I**). Therefore, we consider our results qualitatively robust with respect to sampling, particularly after reweighting with PRE-NMR data.

Note 8: Expansion of the unfolded state on the ribosome

We further considered whether the large conformational expansion observed on the ribosome (26% increase in R_g) could be a consequence of steric exclusion and tethering alone. Therefore, we additionally compared our atomistic ensemble with a simpler MD model where only steric exclusion is considered (see methods). Based on this model, we find an increase in R_g of ~9% from 34.6 ± 0.2 Å to 37.6 ± 0.2 Å relative to the isolated protein (**Extended Data Fig. 5L**) and, also, that the probability of interaction between the C-terminus of FLN5 and the ribosome is decreased as expected (**Extended Data Fig. 5M**). This suggests that the large expansion of the structural ensemble we observed with the RNC is partially (but not fully) caused by tethering and steric exclusion from the ribosome. Other proteins may consequently experience the same effect.

We also note that the structural expansion of the unfolded state on the ribosome (relative to the isolated protein) is also observed prior to reweighting the ensembles with PRE-NMR data. Thus, our all-atom MD simulations also predict the expansion (increase in R_g and solvent-accessible surface area) independent of experiments (**Extended Data Fig. 5N-O**). Moreover, removing the C-terminal datasets (C734, C740, C744), which show the most obvious differences between the isolated and RNC PRE-NMR profiles, does not significantly affect the structural expansion after reweighting (**Extended Data Fig. 5N-O, Supplementary Tables 2-4**).

Note 9: Assumptions and uncertainties involved in estimating solvation entropy and enthalpy changes from the solvent-accessible surface area/MD ensembles

The analysis of SASA/NC hydration to estimate thermodynamic parameters and consequences for folding involves the following main uncertainties and assumptions:

1. The uncertainty in the SASA values must be considered, which is calculated from the MD ensembles (as standard errors of the mean) and reflects the uncertainty due to finite sampling.
2. The empirical parameters used to convert SASA changes to thermodynamic parameters (using equations S25-S28) have an uncertainty themselves. To account for these (parameter) errors, we took an average and standard deviation from values reported in the literature [136] and used standard error propagation to include them in the final results (see Methods).
3. Assessing the potential changes in ribosome surface solvation (and ion binding, see point below) during coTF is currently not possible. We thus assume that the main contributing factor to coTF energetics from solvation arises from changes in NC solvation, without any significant contribution from changes in ribosome surface solvation. Ribosome surface solvation may be influenced by NC-ribosome interactions. This assumption is supported by earlier experiments. Ribosome-NC interactions (and thus ribosome surface solvation) can be effectively modulated by varying the ionic strength (e.g., with arginine/glutamate salt mixtures). We previously found that increasing the ionic strength leads to a slight increase in coTF intermediates and native state on the ribosome by reducing the ribosome interactions and stability of the unfolded state by up to ~ 1 kcal mol^{-1} [6,7]. However, these energetic changes are significantly smaller than the difference we observe between folding on and off the ribosome for FLN5 [6], where unique intermediates are formed and stabilised by more than 4 kcal mol^{-1} on the ribosome. Thus, ribosome interactions and resulting changes in ribosome surface solvation are not sufficient to rationalise the large difference in folding thermodynamics on and off the ribosome. Our data of the FLN5 E6 mutant also shows that strongly reducing the ribosome interactions with the NC is not a major factor in driving the large enthalpy/entropy changes and formation of coTF intermediates (**Extended Data Fig. 7E-H**).
4. We also assume that potential changes in ion (e.g., Mg^{2+}) binding to the ribosome surface during coTF does not have a major effect. This is supported by earlier experiments that showed that the folding free energies on the ribosome are minimally affected by varying the Mg^{2+} concentration [6].

Note 10: Estimating the solvation entropy effects from direct water entropy calculations

To verify the predicted, strong solvation entropy effects, we additionally calculated this quantity using an orthogonal approach. We directly analysed solvent molecules around the unfolded protein and found a ~4% increase in the number of water molecules hydrating the unfolded state on (versus off) the ribosome (**Extended Data Fig. 6J-L**), which was converted into a change in solvation entropy using the two-phase thermodynamic method[137] (see methods). These analyses estimate an entropic

destabilisation of the unfolded NC relative to in isolation, $-T\Delta S_{RNC-iso,solv}$, by ~30 ± 10 kcal mol^{-1} at 298K (**Extended Data Fig. 6M-P**), indicating that the solvent entropy change due to the structural expansion of the unfolded NC is indeed a strong determinant of its altered energetics.

Note 11: Enthalpic destabilisation of the native state on the ribosome

An analysis of NMR ^{1}H, ^{13}C-methyl and ^{19}F chemical shifts of FLN5 RNCs reveals small but widespread chemical shift perturbations (relative to the isolated protein) throughout the protein structure, including sidechains buried in the core (**Extended Data Fig. 7I-J**). Similarly, the ribosome perturbs residue-specific dynamics of methyl sidechains within FLN5 (**Extended Data Fig. 7K**). These data show that the structure and/or the environment of the folded state is altered on the ribosome, despite <1% of ribosome interactions[6,25], which may account for the less negative ΔH of folding on the ribosome. Indeed, factors such as steric exclusion in the vestibule and long-range electrostatic effects between the ribosome surface and negatively charged NCs have previously been suggested to play a role in destabilising native structure on the ribosome[6,9,30,31].

Supplementary tables

**Dataset**	5.5 mg/ml	2.75 mg/ml	1.38 mg/ml	0.69 mg/ml	0.34 mg/ml	0.17 mg/ml	SEC main peak
**R_g (Å)**	32.8 ± 0.6	34.4 ± 0.6	35.9 ± 1.1	35.8 ± 1.6	34.8 ± 1.9	34.9 ± 3.4	35.2 ± 1.2

Table S1. Radii of gyration obtained from individual SAXS datasets at different concentrations (mg/ml) and from the main size exclusion peak (SEC) of FLN5 A_3A_3 V747. The Guinier analysis was performed with the autorg tool of the ATSAS suite.

RNC			
Ensemble	N-term	N-term+ribo	All
Global χ^2	3.93 (6.51)	1.72 (6.51)	1.69 (6.51)
Work χ^2	0.25 (5.78)	2.31 (9.49)	NA
Free χ^2	7.16 (7.15)	0.56 (0.53)	NA
N_{eff}	0.606	0.434	0.411

Table S2. PRE-NMR reweighting statistics (reduced χ^2 and fraction of effective frames, N_{eff}) using different datasets for the FLN5+31 A_3A_3 RNC. The numbers in parentheses show the reduced χ^2 prior to reweighting. Global refers to the agreement with all datasets, work refers to the agreement with the dataset used for reweighting, and free refers to the dataset that was excluded during reweighting. The following lists describe the labelling sites that were included in the individual reweighting datasets:

N-term: C657, C671, C699, C706, C720

N-term+ribo: C657, C671, C699, C706, C720, L23 C90, L24 C53

All: C657, C671, C699, C706, C720, C734, C740, C744, L23 C90, L24 C53

Isolated (τ_C = 3 ns)		
Ensemble	N-term	All
Global χ^2	23.6 (41.5)	3.0 (41.5)
Work χ^2	2.5 (29.2)	NA
Free χ^2	43.9 (53.1)	NA
N_{eff}	0.859	0.684

Table S3. PRE-NMR reweighting statistics (reduced χ^2 and fraction of effective frames, N_{eff}) using different datasets for isolated FLN5 A_3A_3. The numbers in parentheses show the reduced χ^2 prior to reweighting. Global refers to the agreement with all datasets, work refers to the agreement with the dataset used for reweighting, and free refers to the dataset that was excluded during reweighting. The following lists describe the labelling sites that were included in the individual reweighting datasets:

N-term: C657, C699, C706

All: C657, C699, C706, C734, C740, C744

	R_g (Å)		SASA (nm^2)	
Ensemble	Isolated	RNC	Isolated	RNC
Prior	32.1 ± 1.1	45.2 ± 1.9	122.7 ± 1.5	131.8 ± 1.3
N-term	33.2 ± 1.0	44.9 ± 1.7	124.3 ± 1.3	131.9 ± 1.0
N-term+ribo	NA	44.0 ± 2.0	NA	132.0 ± 1.1
All	34.9 ± 1.0	44.1 ± 1.8	126.6 ± 1.1	132.2 ± 1.0

Table S4. Ensemble-averaged R_g and total SASA for isolated FLN5 A_3A_3 and the FLN5+31 A_3A_3 RNC shown before reweighting (prior) and for the different reweighting datasets as outlined in Tables S3 and S4.

**Residue**	**C657**	**C671**	**C699**	**C706**	**C720**	**C734**	**C740**	**C744**	**L23 G90C**	**L24 N53C**
648	4.0	3.6	3.7	4.6	6.5	7.8	54.4	55.0	64.2	34.9
650	3.2	3.6	4.0	3.9	7.8	11.6	39.4	47.2	81.5	32.8
651	3.8	3.7	4.4	4.3	5.4	7.5	21.0	54.5	70.2	19.1
652	3.2	3.6	4.9	4.4	6.6	10.2	28.5	63.8	77.7	22.8
653	3.6	3.9	4.3	4.4	6.6	9.1	26.9	54.8	65.5	17.0
654	3.4	4.0	4.3	6.9	5.4	18.3	23.4	49.2	66.3	22.8
655	3.2	4.1	4.8	5.2	4.7	16.9	26.9	55.2	74.5	25.7
656	3.1	4.6	5.3	5.4	4.9	9.4	30.3	50.1	74.4	29.3
657	3.2	4.6	4.8	5.1	6.0	9.2	35.1	36.0	66.6	27.9
658	3.7	5.1	5.0	5.5	5.0	7.5	40.9	44.3	48.8	28.1

659	3.2	3.9	5.0	4.1	5.5	15.1	36.8	29.5	62.3	17.3
660	4.0	3.3	5.4	7.8	6.6	12.8	38.7	25.2	75.6	14.5
661	3.4	3.8	4.1	5.4	5.6	10.9	57.0	31.8	83.6	16.7
662	3.6	3.9	3.9	4.9	3.9	12.4	76.2	47.3	86.0	10.3
663	4.6	4.0	4.9	6.3	5.5	9.3	83.1	42.9	84.6	8.8
664	3.8	4.3	4.3	7.2	4.7	5.0	83.5	60.8	54.5	8.5
665	3.2	3.8	4.6	6.7	5.1	11.5	100.4	78.6	79.4	16.0
666	3.8	3.8	4.1	4.9	7.6	9.9	112.0	103.2	105.3	30.5
667	3.3	3.4	4.0	4.7	7.8	6.1	99.3	104.2	100.3	46.4
668	4.6	3.7	4.0	5.5	7.6	17.1	108.0	104.0	112.1	54.9
670	6.3	3.5	4.0	5.6	8.9	26.8	104.3	108.4	132.4	74.2
671	5.1	3.2	3.8	4.0	6.5	41.1	98.6	133.0	135.3	78.1
672	5.0	3.9	5.9	3.2	5.7	51.2	68.1	111.6	124.9	77.7
673	4.4	4.1	5.9	5.1	3.8	59.7	94.9	115.0	108.8	83.4
674	4.4	3.8	4.7	6.6	4.4	53.0	81.4	159.3	113.5	96.1
675	4.6	3.6	5.5	5.5	5.3	53.2	64.5	139.4	80.5	62.5
676	3.9	4.7	5.7	4.4	5.2	67.4	59.2	155.7	96.1	89.3
677	4.5	5.7	3.9	4.3	4.9	55.4	43.6	163.5	92.7	91.6
678	6.2	5.0	4.1	4.1	5.8	77.9	60.2	188.3	111.2	68.7
679	5.3	3.8	3.3	5.1	6.2	88.7	49.2	188.2	159.5	87.4
680	4.2	3.8	4.5	4.4	5.6	98.6	30.2	190.1	174.5	123.1
681	4.2	3.5	3.3	4.2	3.5	113.1	60.1	202.3	193.4	119.7
682	4.0	3.2	3.5	4.0	4.3	114.1	76.8	205.5	217.7	113.8
683	3.7	3.8	3.6	3.2	5.0	115.3	77.0	190.4	220.6	96.0
684	3.8	4.3	3.4	3.5	6.0	110.8	58.1	185.5	189.5	113.2
685	4.7	4.1	3.3	3.4	7.4	95.8	91.2	193.1	186.5	120.5
686	3.7	4.8	3.9	4.1	8.5	66.4	96.4	169.5	154.4	111.1
687	3.8	3.7	3.6	4.5	5.5	41.9	96.3	154.2	116.4	102.2
688	4.1	3.5	3.9	5.5	4.7	22.2	98.5	137.9	143.9	100.3

689	3.3	3.9	4.2	3.8	4.9	32.5	105.3	160.9	156.0	113.7
691	3.6	4.1	4.1	3.5	5.5	53.4	129.4	189.4	189.4	114.1
692	5.8	3.8	5.0	3.7	7.0	98.4	131.9	195.4	216.4	101.6
693	6.5	4.2	5.4	3.8	5.6	100.9	135.1	212.9	241.6	113.3
694	6.6	4.0	5.0	3.2	4.6	57.7	136.9	214.6	247.4	94.5
695	7.1	3.6	3.2	4.1	4.4	65.9	139.3	198.2	260.5	92.7
696	5.6	4.0	5.8	3.3	3.7	81.7	136.2	195.7	257.8	103.3
697	4.3	3.6	5.5	3.9	4.5	75.9	116.2	163.0	224.5	90.2
699	3.4	4.8	5.6	4.7	4.5	82.7	119.3	174.3	225.5	92.3
700	5.3	4.5	3.8	8.5	6.0	35.4	97.2	58.7	166.7	33.8
701	4.8	4.0	3.6	11.3	5.3	69.1	93.8	64.7	156.8	45.0
702	4.6	4.1	4.4	10.3	6.9	63.9	109.2	124.1	161.9	23.8
703	4.3	3.8	8.3	13.0	4.7	40.8	127.1	211.3	125.8	65.1
704	4.4	3.5	9.7	16.6	7.4	101.0	145.4	243.4	150.0	70.6
705	4.3	5.8	5.5	15.5	5.9	90.4	90.1	219.6	155.5	105.9
706	3.2	4.0	8.4	10.3	8.7	76.6	77.5	249.7	145.9	142.0
707	3.6	3.8	8.8	3.6	7.1	74.5	76.8	265.6	175.9	133.1
708	4.1	4.3	9.5	3.8	8.3	80.5	103.4	236.0	123.5	141.2
709	6.7	5.2	8.2	8.4	7.4	44.3	107.7	271.8	76.3	109.6
710	6.5	5.6	10.2	13.1	15.4	60.5	103.7	275.6	99.8	109.5
711	5.4	5.7	10.0	14.4	23.2	38.8	61.0	229.1	93.1	89.6
712	3.6	6.2	7.1	19.8	5.4	20.8	69.7	223.3	77.0	84.6
713	5.0	3.9	7.9	17.2	23.9	29.4	61.8	212.0	78.2	97.0
714	7.8	3.9	5.0	15.8	25.4	13.3	25.4	155.2	88.9	76.1
715	7.0	3.4	4.2	8.8	23.5	15.4	6.8	126.4	72.8	58.9
716	8.1	4.0	3.9	7.1	25.1	26.7	8.6	90.5	111.5	34.0
717	5.2	3.9	4.2	5.8	28.0	26.6	5.7	38.0	88.0	7.3
718	5.2	4.0	3.8	3.6	40.7	31.1	7.0	47.5	117.5	13.4
719	7.1	4.3	3.5	5.3	40.5	7.8	5.0	109.6	164.3	37.2

720	7.6	3.8	3.5	4.9	33.7	14.6	31.3	168.6	193.6	8.3
721	6.2	4.4	5.6	9.5	8.0	12.3	36.9	153.0	164.2	11.1
723	5.4	5.0	7.1	8.8	5.4	7.2	59.0	182.5	180.1	16.8
724	4.5	6.4	5.7	12.5	50.7	15.5	23.2	113.1	173.3	6.0

Table S5. Prior RNC ensemble residue- and MTSL-labelling site specific effective τ_C values for all non-proline residues in the NMR-visible region of the FLN5 A_3A_3 sequence calculated using equation S16 (see methods).

Residue	C657	C671	C699	C706	C720	C734	C740	C744	L23 G90C	L24 N53C
648	3.9	13.4	4.6	11.1	4.9	6.7	53.9	42.4	23.2	36.4
650	3.7	10.5	3.8	4.7	6.6	10.9	40.7	36.1	16.1	56.9
651	11.8	4.6	4.0	5.8	5.6	15.8	18.7	28.0	19.9	37.2
652	8.6	4.0	4.0	4.8	7.6	19.5	25.6	39.1	22.3	32.2
653	5.3	3.8	3.6	4.0	13.3	20.1	21.0	31.9	23.7	20.3
654	3.4	3.9	3.8	6.6	8.3	24.7	13.7	25.7	27.1	28.3
655	4.3	4.0	4.8	4.8	6.9	23.1	19.3	24.0	28.9	21.0
656	5.2	4.5	8.3	6.8	4.9	14.7	20.5	29.4	29.5	20.2
657	5.0	4.7	8.3	6.4	4.3	17.4	24.5	25.0	22.0	19.2
658	5.8	5.2	9.7	7.3	4.0	12.6	26.4	32.7	19.9	18.2
659	4.4	5.6	8.9	4.2	4.4	10.4	29.3	28.0	24.7	15.5
660	4.6	3.6	10.1	6.3	5.1	10.3	32.6	22.6	20.7	12.2
661	4.5	4.3	6.2	5.8	4.1	7.1	38.9	22.8	17.0	12.4
662	4.9	5.9	4.7	5.6	3.6	10.0	46.1	26.0	13.6	7.7
663	7.1	5.2	5.2	5.7	4.0	6.4	41.1	18.9	16.7	8.5
664	3.9	5.8	5.4	6.0	3.7	7.5	36.7	30.0	21.4	12.3
665	3.2	6.5	5.8	5.7	4.0	10.3	49.7	48.2	20.8	18.0
666	3.3	5.3	5.4	4.5	5.1	6.4	55.9	64.7	20.3	23.6
667	3.4	5.8	4.6	4.1	3.8	5.9	55.0	67.8	26.0	31.7
668	3.7	5.9	4.1	5.5	4.8	11.8	75.0	74.7	32.2	44.5
670	4.2	5.2	5.3	5.4	7.1	17.9	81.1	89.4	39.1	46.9

671	5.2	5.6	3.9	6.9	5.6	23.2	64.8	79.4	46.7	25.3
672	5.4	10.2	5.1	5.1	7.3	27.5	65.2	81.0	32.5	22.2
673	4.8	8.3	4.3	5.3	5.3	23.1	61.3	81.5	31.5	19.2
674	6.6	4.6	5.0	6.1	5.7	19.4	60.2	96.7	42.6	22.9
675	8.0	8.5	4.6	13.1	7.5	21.8	47.9	91.8	33.3	26.1
676	5.8	6.0	5.7	5.9	5.6	24.5	37.1	96.5	36.0	37.6
677	5.5	7.5	3.4	4.7	5.4	26.9	23.2	97.6	40.7	43.3
678	6.8	5.1	3.5	3.5	7.7	37.6	41.8	126.6	40.5	40.8
679	5.4	5.1	3.3	4.6	5.4	43.0	29.2	128.4	59.5	54.8
680	4.1	5.0	4.4	5.5	6.4	44.4	13.1	120.7	58.0	77.9
681	4.9	5.5	5.6	4.7	9.7	44.8	17.0	120.9	58.3	65.1
682	4.3	5.3	5.8	5.3	6.4	45.8	38.8	131.5	60.9	63.8
683	4.0	4.7	9.8	3.8	4.7	44.7	67.9	125.3	59.6	61.0
684	4.5	6.0	4.1	4.3	6.7	46.9	53.7	128.4	41.4	76.2
685	4.1	5.5	3.8	4.3	6.9	30.6	64.8	127.3	43.3	85.3
686	3.2	7.5	4.7	4.8	8.4	13.9	67.2	91.0	40.7	65.0
687	3.5	5.5	5.6	10.7	6.5	7.6	58.5	78.5	43.9	56.5
688	4.1	4.2	4.6	4.2	6.7	3.9	40.9	59.9	62.6	45.7
689	3.5	4.3	4.0	4.7	5.3	5.6	42.1	70.9	62.5	56.0
691	4.1	5.7	4.8	3.5	4.8	17.7	60.1	91.3	66.9	66.3
692	6.3	8.2	8.9	4.0	6.8	29.4	61.5	86.5	86.0	50.1
693	4.8	14.9	9.1	4.2	4.1	38.0	70.4	119.2	82.6	77.5
694	5.5	9.5	12.2	3.4	3.2	23.2	68.3	123.2	92.9	71.2
695	5.3	4.8	8.4	4.1	5.0	23.5	76.8	98.4	95.0	45.0
696	6.5	4.3	12.7	5.1	4.6	35.8	81.5	105.5	90.1	39.9
697	5.9	3.9	12.2	5.4	4.8	37.1	74.9	90.7	55.8	38.2
699	4.0	4.1	15.3	3.7	6.6	39.1	68.1	68.5	77.5	23.3
700	10.4	4.1	13.0	6.2	10.4	14.0	49.9	23.3	61.1	15.9
701	6.1	3.9	10.0	12.2	9.6	21.9	37.8	21.0	47.4	10.4

702	5.6	3.3	13.8	14.7	11.3	16.1	47.9	26.4	57.0	9.8
703	4.5	3.2	10.2	10.9	6.8	7.7	64.2	65.3	53.3	20.2
704	3.6	3.4	10.4	15.1	16.7	15.3	96.4	104.8	57.3	24.9
705	3.9	5.9	5.9	12.5	10.9	13.9	105.1	144.1	37.6	42.9
706	4.1	5.3	8.3	7.4	8.2	14.4	113.9	188.7	25.4	51.4
707	5.8	4.1	8.2	5.7	4.9	52.6	128.5	217.8	41.0	41.2
708	5.9	4.9	8.0	8.3	7.0	81.4	125.1	224.7	37.2	62.7
709	8.7	6.9	9.3	7.7	10.0	70.3	104.6	225.5	15.6	50.5
710	7.0	9.6	8.5	9.4	12.9	100.3	92.5	225.9	42.8	56.8
711	4.7	9.2	7.1	9.8	22.0	85.1	54.6	183.0	40.7	62.2
712	3.9	10.9	8.8	14.3	10.6	83.9	94.9	218.4	38.9	70.4
713	4.6	11.4	6.3	14.1	19.8	83.9	89.6	206.8	27.8	71.0
714	6.7	5.0	7.3	22.6	21.3	62.5	63.3	176.9	37.1	51.9
715	5.5	4.8	5.3	15.4	21.6	47.0	45.5	172.7	29.9	55.3
716	6.4	4.1	4.6	8.3	21.3	46.5	82.3	111.0	31.3	37.2
717	4.3	3.2	5.6	10.9	23.1	35.5	40.5	104.3	28.7	5.2
718	3.9	3.3	4.1	12.2	29.7	33.1	53.4	126.9	28.7	11.7
719	5.9	5.7	3.6	8.3	36.4	6.4	60.0	202.8	55.3	49.7
720	6.3	3.8	5.3	6.7	27.4	18.3	121.5	286.8	71.1	28.8
721	4.3	5.5	10.0	9.3	17.9	16.9	94.5	271.3	82.1	21.3
723	4.3	7.3	13.0	12.9	13.1	12.6	68.7	291.6	160.1	24.1
724	4.2	10.9	11.5	17.4	33.3	17.6	39.6	230.1	146.9	4.8

Table S6. Posterior RNC ensemble (reweighted with all ten labelling sites) residue- and MTSL-labelling site specific effective τ_C values for all non-proline residues in the NMR-visible region of the FLN5 A_3A_3 sequence calculated using equation S16 (see methods).

Residue	FLN5+31 A_3A_3	FLN5+47 A_3A_3	FLN5+67 A_3A_3	Isolated FLN5 A_3A_3
E724	0.31 ± 0.20	0.44 ± 0.13	0.41 ± 0.14	0.27 ± 0.02
A727	n.m.	0.45 ± 0.21	0.43 ± 0.17	0.10 ± 0.03
N728	n.m.	0.20 ± 0.53	0.27 ± 0.32	0.21 ± 0.05

Table S7. PRE-NMR intensity ratios for C-terminal residues of isolated and RNC FLN5 A_3A_3 samples labelled at position C740 with MTSL. These residues are close to the ribosome-interacting region (residues 730-750) and support the notion that the C-terminal region of residues 730-750 (not quantifiable by NMR due to ribosome interactions) is more expanded on the ribosome than in isolation.

**1. Convergence of simulations and analysis**
**Is an evaluation presented in the text to show that the property being measured has equilibrated in the simulations (e.g., time-course analysis)?**
Yes, see supplementary note 7, supplementary figures 5 & 8.
**Then, is it described in the text how simulations are split into equilibration and production runs and how much data were analyzed from production runs? Are there at least 3 simulations per simulation condition with statistical analysis?**
Described in the Methods (10x independent simulations on and off the ribosome to generate the conformational ensembles).
**Is evidence provided in the text that the simulation results presented are independent of initial configuration?**
Yes, see supplementary note 7, supplementary figures 5 & 8.
**2. Connection to experiments**
**Are calculations provided that can connect to experiments (e.g., loss or gain in function from mutagenesis, binding assays, NMR chemical shifts, J-couplings, SAXS curves, interaction distances or FRET distances, structure factors, diffusion coefficients, bulk modulus and other mechanical properties, etc.)?**
Yes, see Extended Data Fig. 3-5 and supplementary notes 5-7. We compared simulations to PRE-NMR data (used for reweighting), NMR chemical shifts, RDCs, NMR diffusion (radius of hydration), and SAXS.
**3. Methods choice**
**Do simulations contain membranes, membrane proteins, intrinsically disordered proteins, glycans, nucleic acids, polymers, or cryptic ligand binding?**
Yes, intrinsically disordered proteins (unfolded state ensembles) and nucleic acids.
**Is it described in the text whether the accuracy of the chosen model(s) is sufficient to address the question(s) under investigation (e.g., all-atom vs. coarse-grained models, fixed charge vs. polarizable force fields, implicit vs. explicit solvent or membrane, specific force field and water model, etc.?**
Yes, we have compared different force fields with experimental data for the unfolded state ensemble in isolation (supplementary notes 5-6). For the ribosome-nascent chain complex, we have also compared the simulations to NMR measurements quantifying nascent chain interactions with the ribosome surface and found good agreement with experiments (Extended Data Fig. 5G-H).
**Is the timescale of the event(s) under investigation beyond the brute-force MD simulation timescale in this study that enhanced sampling methods are needed?**
No.
**If NO, is the evidence provided in the text?**
Yes, see supplementary note 7, supplementary figures 5 & 8.
**4. Code and reproducibility**

**Is a table provided describing the system setup that includes simulation box dimensions, total number of atoms, number of water molecules, salt concentration, lipid composition (number of molecules and type)?**
Described in the Methods.
**Are other parameters for the system setup described in the text, such as protonation state, type of structural restraints if applied, nonbonded cutoff, thermostat and barostat, etc.?**
Described in the Methods.
**Is it described in the text what simulation and analysis software and which versions are used?**
Described in the Methods and Reporting Summary.
**Are initial coordinate and simulation input files and a coordinate file of the final output provided as supplementary files or in a public repository?**
Initial coordinate files, topology files, simulation input files, and trajectories containing all protein atoms are deposited on Zenodo (see Data Availability).
**Is there custom code or custom force field parameters?**
No.

Table S8. Reliability and reproducibility checklist for the molecular dynamics data (https://www.nature.com/articles/s42003-023-04653-0/tables/1).

Supplementary figures

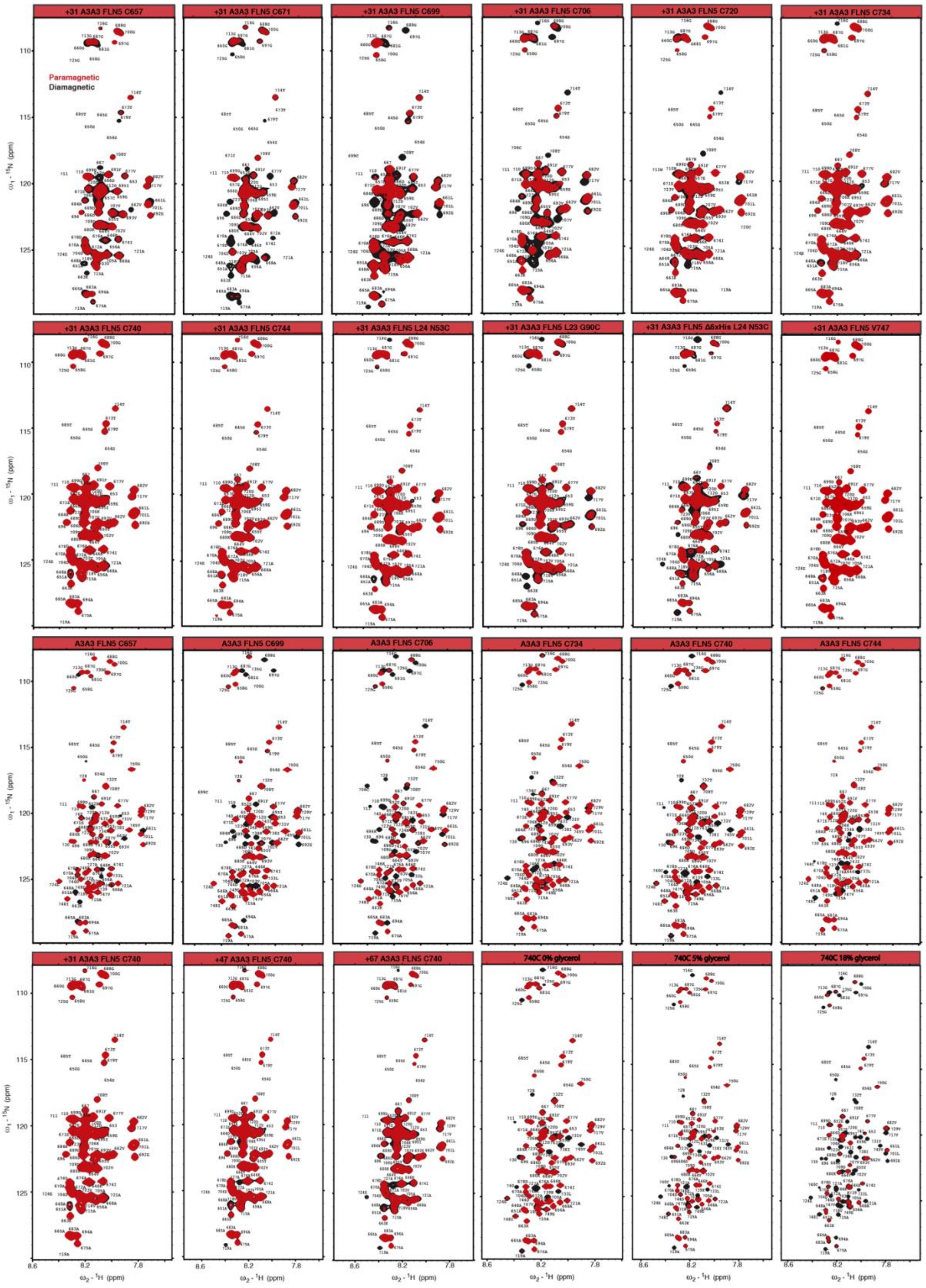

Fig. S1: ^{1}H-^{15}N SOFAST-HMQC PRE-NMR spectra of isolated and RNC variants. The paramagnetic (red) and diamagnetic (grey) spectra are overlaid, and residue assignments labelled. All spectra were recorded at 800MHz, 283K.

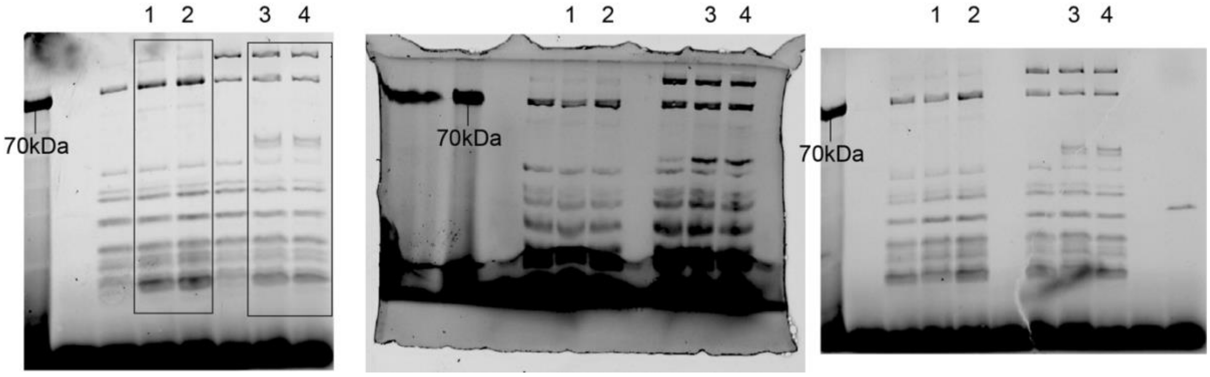

Fig. S2: Raw gel images of ABD-MTS reactions with ribosomes and RNCs. The rectangular boxes show the cropped area of the gel presented in the Extended Data. (1,2) 70S ribosomes reacted with ABD-MTS for 30 and 60 minutes, respectively. (3,4) FLN5+31 A3A3 C699 RNC reacted with ABD-MTS for 30 and 60 minutes, respectively.

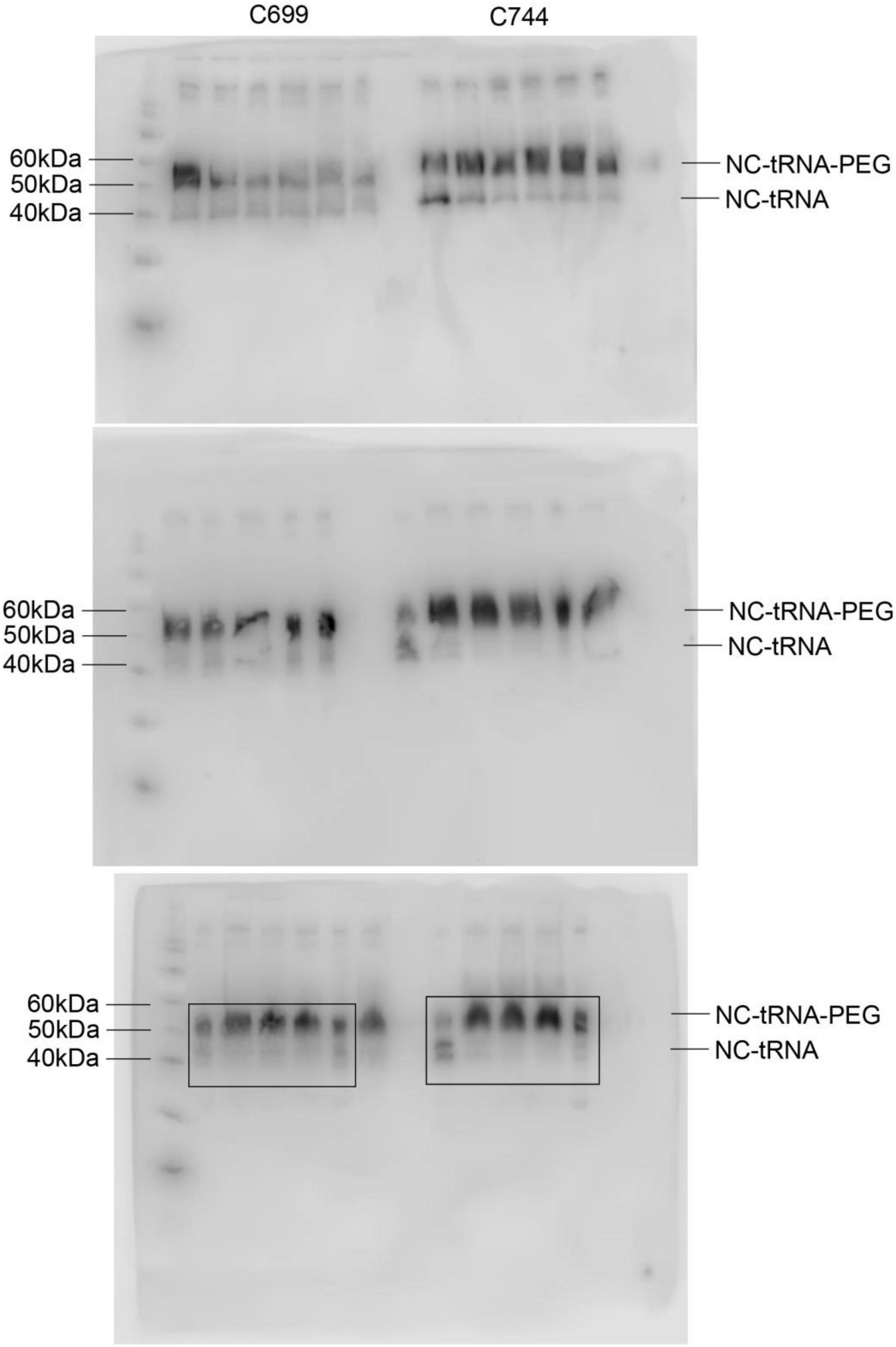

Fig. S3: Raw western blot images of FLN5+31 A3A3 V747 RNCs labelled with PEG-maleimide at different cysteine positions. The rectangular boxes show the cropped area of the gel presented in the Extended Data.

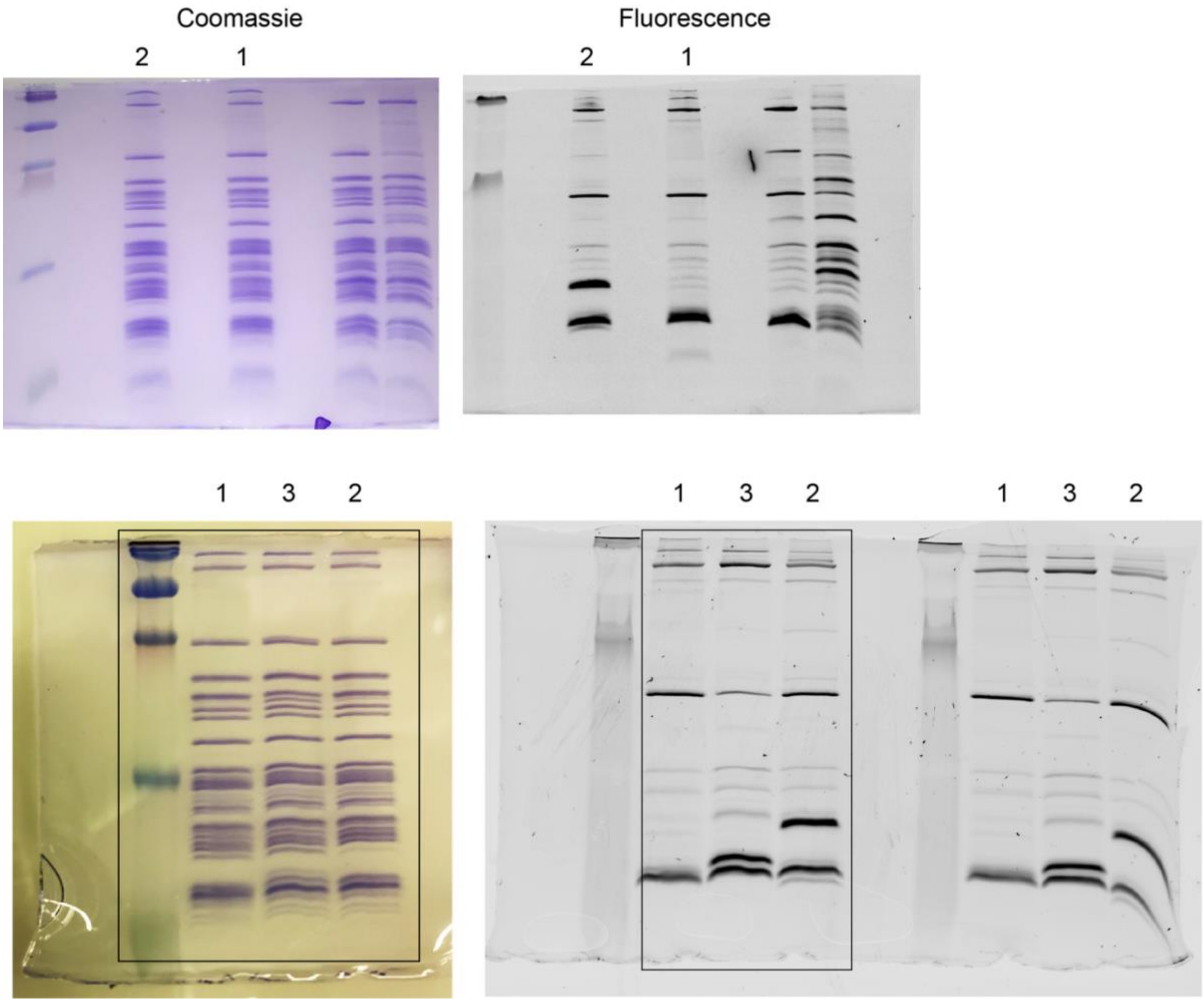

Fig. S4: Raw Coomassie and fluorescne gel images of ribosomes reacted with fluorescein maleimide. The rectangular boxes show the cropped area of the gel presented in the Extended Data. (1) WT 70S ribosomes; (2) uL24 N53C 70S ribosomes; (3) uL23 G90C 70S ribosomes.

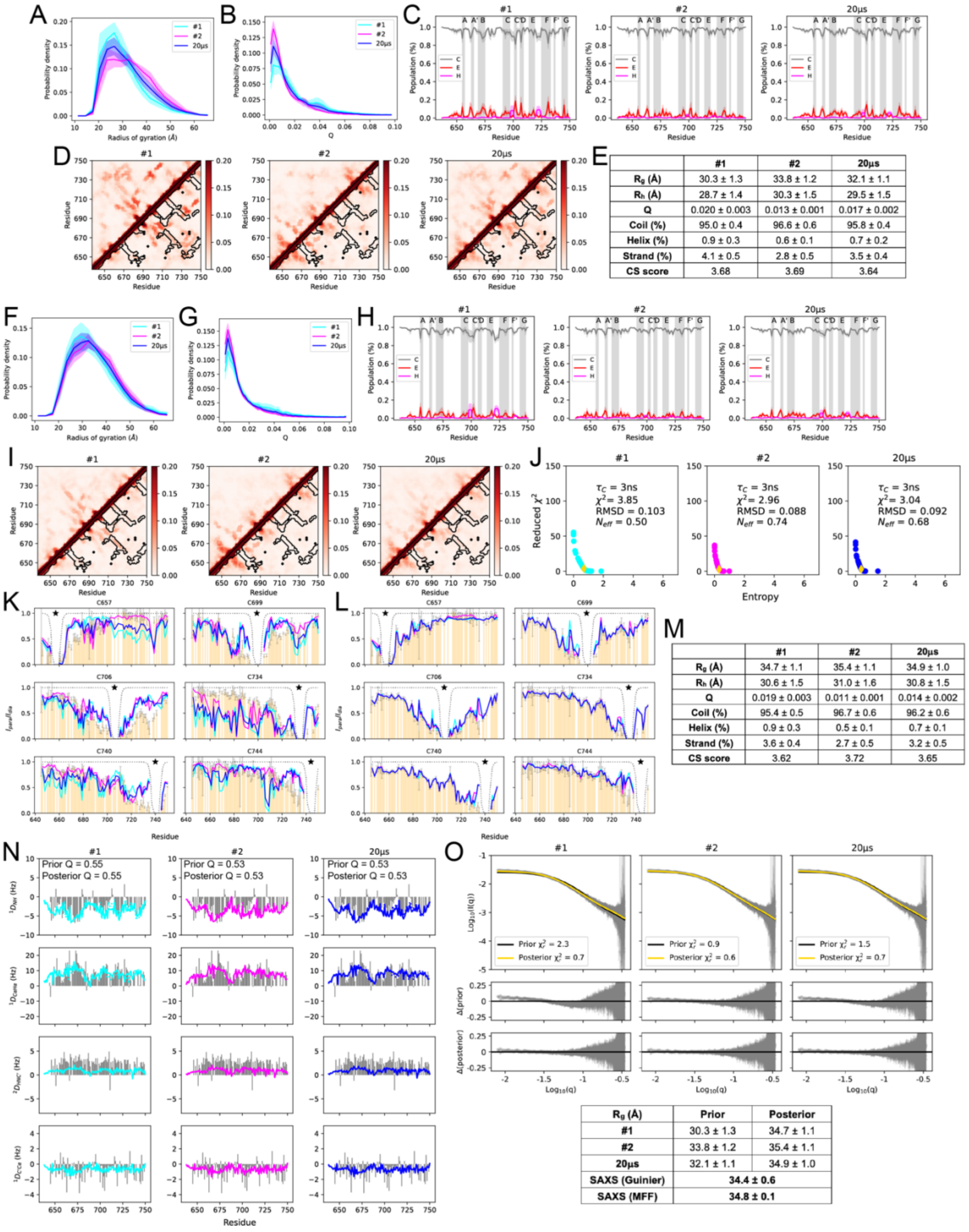

	#1	#2	20μs
R_g (Å)	30.3 ± 1.3	33.8 ± 1.2	32.1 ± 1.1
R_h (Å)	28.7 ± 1.4	30.3 ± 1.5	29.5 ± 1.5
Q	0.020 ± 0.003	0.013 ± 0.001	0.017 ± 0.002
Coil (%)	95.0 ± 0.4	96.6 ± 0.6	95.8 ± 0.4
Helix (%)	0.9 ± 0.3	0.6 ± 0.1	0.7 ± 0.2
Strand (%)	4.1 ± 0.5	2.8 ± 0.5	3.5 ± 0.4
CS score	3.68	3.69	3.64

	#1	#2	20μs
R_g (Å)	34.7 ± 1.1	35.4 ± 1.1	34.9 ± 1.0
R_h (Å)	30.6 ± 1.5	31.0 ± 1.6	30.8 ± 1.5
Q	0.019 ± 0.003	0.011 ± 0.001	0.014 ± 0.002
Coil (%)	95.4 ± 0.5	96.7 ± 0.6	96.2 ± 0.6
Helix (%)	0.9 ± 0.3	0.5 ± 0.1	0.7 ± 0.1
Strand (%)	3.6 ± 0.4	2.7 ± 0.5	3.2 ± 0.5
CS score	3.62	3.72	3.65

R_g (Å)	Prior	Posterior
#1	30.3 ± 1.3	34.7 ± 1.1
#2	33.8 ± 1.2	35.4 ± 1.1
20μs	32.1 ± 1.1	34.9 ± 1.0
SAXS (Guinier)	34.4 ± 0.6	
SAXS (MFF)	34.8 ± 0.1	

Fig. S5: Convergence analysis of the C36m+W ensemble by comparing the first (#1) and second (#2) sets of 10μs and the full 20μs ensemble. MD-calculated properties are shown as the mean ± SEM obtained from block averaging. **(A)** R_g probability distributions of the ensembles before reweighting. **(B)** Native contact fraction (Q) probability distributions of the ensembles before before reweighting. **(C)** Secondary structure profiles along with the annotated native strands before reweighting. **(D)** Contact maps overlayed with the native contact map in black contours before reweighting. **(E)** Average properties ensembles before reweighting including the chemical shift score, quantifying the agreement with chemical shifts (see Methods). **(F)** Comparison of the C36m+W ensembles

after reweighting using the probability distribution of R_g and fraction of native contacts **(G)**. **(H)** Secondary structure profiles along with the annotated native strands after reweighting. **(I)** Contact maps after reweighting overlayed with the native contact map in black contours. **(J)** L-curve analysis of the ensembles when reweighted with the PRE-NMR data. The final statistics obtained at the elbow point of the curve are annotated on the plot. **(K)** Comparison of the calculated PREs from the ensembles (same colour scheme as in panel J) with the experimental data (orange) before and after **(L)** reweighting. **(M)** Average properties of the reweighted C36m+W ensembles including the chemical shift score, quantifying the agreement with chemical shifts (see methods). **(N)** The simulated RDCs of the C36m+W ensembles before (dotted line) and after reweighting (solid line) with the PRE-NMR data are shown. The experimental RDCs, measured in PEG/Octanol, are shown in grey bars. **(O)** Comparison of the experimental and theoretical SAXS profiles obtained from the C36m+W ensembles before and after reweighting. The goodness of fit is quantified with the reduced χ^2 and residuals are shown below the main plot for the prior and posterior ensembles. The table shows the average R_g from the ensembles compared to the SAXS-derived experimental values by Guinier and molecular form factor (MFF) analysis.

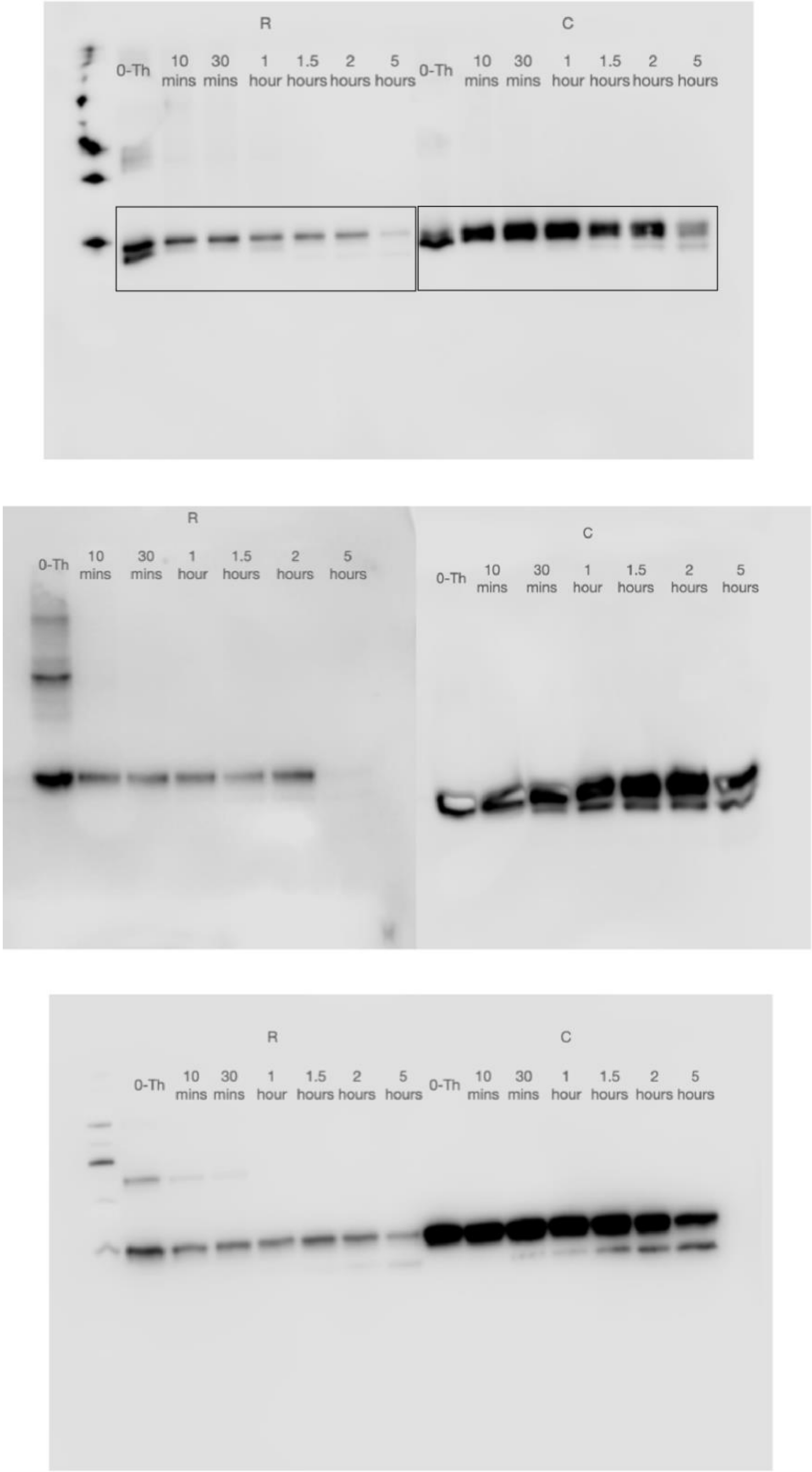

Fig. S6: Raw western blot images of HRAS refolding experiments *in vitro*. Refolded (R) and control (C) samples were subjected to pulse proteolysis (proteolysis times annotated). The rectangular boxes show the cropped area of the gel presented in the Extended Data.

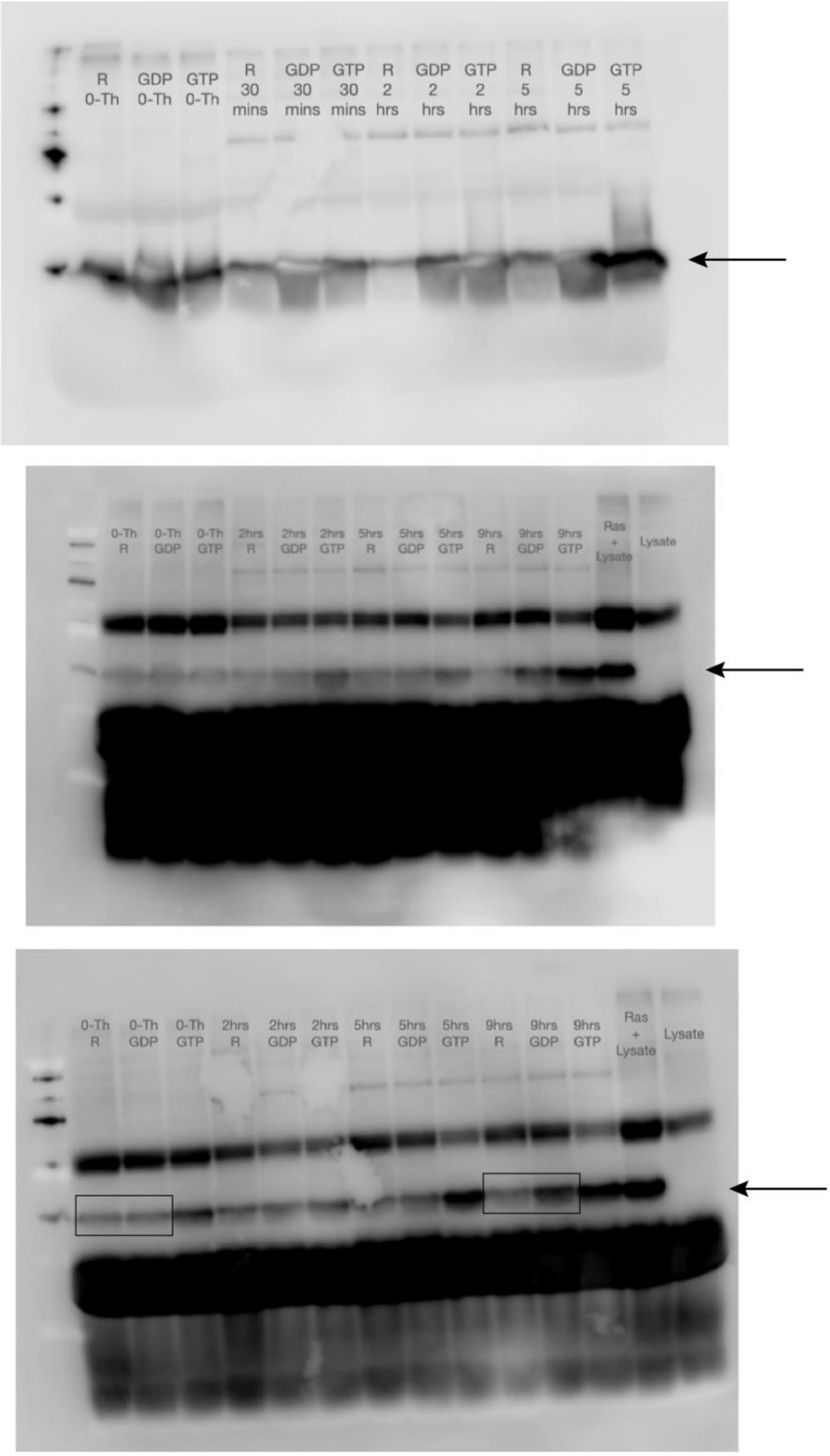

Fig. S7: Raw western blot images of HRAS refolding experiments in rabbit reticulocyte lysate (RRL). Refolded (R) and control (labelled with 'GDP') samples were subjected to pulse proteolysis (proteolysis times annotated). The arrows point to HRAS on the images and the rectangular boxes show the cropped area of the gel presented in the Extended Data.

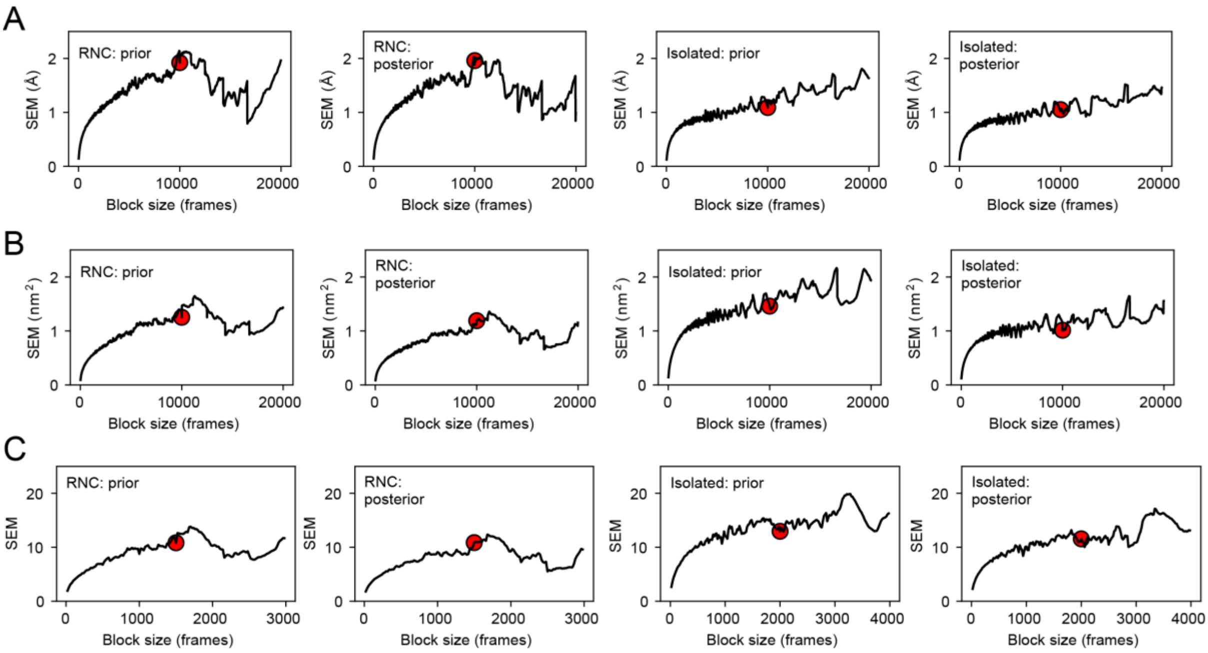

Fig. S8: (A-C) Block analysis to estimate the standard error of the mean (SEM) for the isolated and RNC ensemble properties including R_g **(A)**, SASA **(B)** and number of water molecules in the hydration layer **(C)** before (prior) and after (posterior) reweighting with PRE-NMR data. The block size is shown in number of frames. The total number of frames in the ensemble is 100,000 for each ensemble (except for the analysis for the number of water molecules in the hydration layer; the RNC and isolated ensembles contain 15,000 and 20,000 frames, respectively, due to saving water coordinates only every 1ns). The red point shows the block size chosen to calculate the final error. Note that we chose a block size equal to 10% of the full ensemble due to having run 10 independent simulations for each system. Thus, these ten statistically independent simulations are used to estimate the SEM.

References

143 Dedmon, M. M., Lindorff-Larsen, K., Christodoulou, J., Vendruscolo, M. & Dobson, C. M. Mapping long-range interactions in alpha-synuclein using spin-label NMR and ensemble molecular dynamics simulations. *J Am Chem Soc* **127**, 476-477, doi:10.1021/ja044834j (2005).

144 Lindorff-Larsen, K. *et al.* Determination of an ensemble of structures representing the denatured state of the bovine acyl-coenzyme a binding protein. *J Am Chem Soc* **126**, 3291-3299, doi:10.1021/ja039250g (2004).

145 Allison, J. R., Varnai, P., Dobson, C. M. & Vendruscolo, M. Determination of the free energy landscape of alpha-synuclein using spin label nuclear magnetic resonance measurements. *J Am Chem Soc* **131**, 18314-18326, doi:10.1021/ja904716h (2009).

146 Deckert, A. *et al.* Structural characterization of the interaction of alpha-synuclein nascent chains with the ribosomal surface and trigger factor. *Proc Natl Acad Sci U S A* **113**, 5012-5017, doi:10.1073/pnas.1519124113 (2016).

147 Clore, G. M. & Iwahara, J. Theory, practice, and applications of paramagnetic relaxation enhancement for the characterization of transient low-population states of biological macromolecules and their complexes. *Chem Rev* **109**, 4108-4139, doi:10.1021/cr900033p (2009).

148 Gillespie, J. R. & Shortle, D. Characterization of long-range structure in the denatured state of staphylococcal nuclease. I. Paramagnetic relaxation enhancement by nitroxide spin labels. *J Mol Biol* **268**, 158-169, doi:10.1006/jmbi.1997.0954 (1997).

149 Battiste, J. L. & Wagner, G. Utilization of site-directed spin labeling and high-resolution heteronuclear nuclear magnetic resonance for global fold determination of large proteins with limited nuclear overhauser effect data. *Biochemistry* **39**, 5355-5365, doi:10.1021/bi000060h (2000).

150 Chan-Yao-Chong, M. *et al.* Structural Characterization of N-WASP Domain V Using MD Simulations with NMR and SAXS Data. *Biophys J* **116**, 1216-1227, doi:10.1016/j.bpj.2019.02.015 (2019).

151 Lindorff-Larsen, K., Trbovic, N., Maragakis, P., Piana, S. & Shaw, D. E. Structure and dynamics of an unfolded protein examined by molecular dynamics simulation. *J Am Chem Soc* **134**, 3787-3791, doi:10.1021/ja209931w (2012).
